# Earlier onset of type 2 diabetes mellitus as a risk factor for Irvine-Gass syndrome

**DOI:** 10.1016/j.clinsp.2026.101204

**Published:** 2026-10-14

**Authors:** Yingxia Yin, Yu Du, Junyi Wang, Wan Lu, Shancheng Si

**Affiliations:** a Department of Ophthalmology, Beijing Geriatric Hospital, Beijing, China; b Beijing Tsinghua Changgung Hospital Eye Center, Beijing Visual Science and Translational Eye Research Institute (BERI), Tsinghua Medicine, Tsinghua University, Beijing, China

**Keywords:** Irvine-Gass Syndrome, Cystoid macular edema, Early-onset type 2 diabetes mellitus

## Abstract

**Purpose:**

To investigate the association between age at type 2 diabetes mellitus (T2DM) onset and the risk of Irvine-Gass Syndrome (IGS) following uneventful phacoemulsification with intraocular lens implantation (PEI).

**Methods:**

This retrospective case-control study analyzed 190 participants (39 IGS, 151 non-IGS) who underwent uneventful PEI. Comprehensive demographic, systemic, and perioperative data were collected. Statistical analyses included between-group comparisons, logistic regression, and Receiver Operating Characteristic (ROC) curve analysis to identify independent IGS predictors and determine optimal risk-stratification thresholds.

**Results:**

While overall T2DM prevalence was comparable between groups (p = 0.168), IGS patients had significantly earlier diabetes onset (49.05±8.61 vs. 59.23±10.65 years, p < 0.001). Multivariable analysis confirmed younger T2DM onset age as an independent IGS predictor (OR = 1.094 per year decrease, 95% Confidence Interval [95% CI 1.015‒1.179], p = 0.019). ROC analysis identified T2DM onset age ≤ 55.5-years as the optimal predictive threshold (Area Under the ROC Curve [AUROC = 0.774], sensitivity = 73.7%, specificity = 78.9%). Participants stratified by this threshold showed significantly different IGS incidence (non-diabetic: 15.32%; younger-onset T2DM: 56.67%; older-onset T2DM: 10.20%; p < 0.001). Younger-onset T2DM patients had approximately 5.5-fold higher IGS odds versus non-diabetics (adjusted OR = 5.499, 95% CI 2.104‒14.373, p = 0.001).

**Conclusions:**

Earlier T2DM onset (≤ 55.5-years) is a strong independent risk factor for IGS following uneventful PEI. This threshold provides a clinically useful tool for preoperative risk stratification, particularly valuable for identifying high-risk diabetic patients who may benefit from enhanced perioperative monitoring and management.

## Highlights

• Younger-onset T2DM (≤ 55.5-years) as a risk factor for Irvine-Gass syndrome.
• Threshold of T2DM onset ≤ 55.5-years: 73.7% sensitivity, 78.9% specificity.
• Early metabolic disease duration disrupts blood-retinal barrier integrity.

## Introduction

Pseudophakic Cystoid Macular Edema (CME), first described by Irvine and Gass, is a well-known cause of painless visual impairment following cataract surgery.^1^, ^2^, ^3^ This condition, now commonly referred to as Irvine-Gass Syndrome (IGS), is characterized by perifoveal capillary leakage and intraretinal fluid accumulation, typically detectable on Optical Coherence Tomography (OCT).^1^^,^^2^ While the pathogenesis is considered multifactorial, the disruption of the blood-retinal barrier due to surgical trauma and subsequent inflammatory mediation ‒ notably involving prostaglandins ‒ is a central mechanism.^2^^,^^3^

The reported incidence of IGS varies considerably, with recent studies citing a rate of 2.3%‒5% for clinically symptomatic, OCT-confirmed CME after uneventful phacoemulsification with intraocular lens implantation (PEI).^1^^,^^2^^,^^4^ However, this risk is not uniform across all patient groups. Several predisposing factors have been established, including a history of Diabetic Retinopathy (DR), retinal vein occlusion, epiretinal membrane, uveitis, and intraoperative complications such as posterior capsule rupture.^2^, ^3^, ^4^, ^5^, ^6^, ^7^ The role of diabetes mellitus itself is particularly noteworthy. Kim et al. reported a strikingly high IGS incidence of 22% in diabetic patients, underscoring diabetes as a major risk factor.^8^ Furthermore, the preoperative use of prostaglandin analogues for glaucoma have also been implicated.^2^^,^^5^

In the context of a global surge in Type 2 Diabetes Mellitus (T2DM), a new and concerning epidemiological trend has emerged: a sharp increase in early-onset T2DM among individuals under 40-years of age.^9^ This patient subgroup is clinically distinct, often exhibiting a more aggressive disease course and a higher propensity for microvascular complications due to prolonged exposure to metabolic dysregulation.^9^ This observation led us to a critical, yet unexamined, hypothesis: Could the age at T2DM onset, rather than merely its presence, be a key determinant of IGS risk?

Our clinical experience suggested that not all diabetic patients carry an equal risk for IGS. Specifically, we noted that patients who developed T2DM at a younger age appeared disproportionately represented among IGS cases following otherwise uneventful surgery. While previous large-scale studies, such as the IRIS® Registry analysis, have confirmed younger patient age as a general risk factor for CME, they have not dissected the specific contribution of early-onset T2DM from chronological age itself.^10^ This represents a significant knowledge gap. The precise relationship between the age of T2DM onset and the risk of IGS, particularly in patients without confounding ocular comorbidities, remains unexplored.

Therefore, the primary aim of this case-control study was to investigate the association between the age at T2DM onset and the risk of developing clinical IGS after uneventful PEI. We rigorously excluded patients with known ocular risk factors (e.g., DR, prior uveitis, retinal vascular diseases) and surgical complications to isolate the systemic effect of T2DM. A secondary aim was to evaluate other potential systemic risk factors, including systemic inflammatory markers such as lipid profiles and platelet counts, which have been preliminarily linked to inflammatory states and blood-retinal barrier integrity.^11^, ^12^, ^13^ By identifying a specific, high-risk diabetic phenotype, this research seeks to provide a refined tool for preoperative stratification and pave the way for more personalized prophylactic strategies.

## Materials and methods

### Participants and perioperative management

A retrospective case-control study was conducted between June 2022 and January 2025, involving patients who underwent uneventful PEI (defined as a procedure without posterior capsule rupture, vitreous loss, significant corneal edema, or other intraoperative complications), with or without subsequent IGS. The study protocol received approval (n° 25,586-6–01) from the Institutional Review Board and adhered to the principles outlined in the Declaration of Helsinki. Due to the retrospective design of the study, the review board granted a waiver for written informed consent. All patient data were anonymized and de-identified.

All PEI surgeries were performed under topical anesthesia by a Single experienced Surgeon (S.S.). A standardized postoperative medication regimen was uniformly adopted for all patients. This consisted of a broad-spectrum topical antibiotic (levofloxacin 0.5%) administered four times daily for 2-weeks, combined with a topical corticosteroid (prednisolone acetate 1%) administered four times daily and tapered over 4-weeks.

All patients were scheduled for follow-up examinations at 1-day, 1-week, 1-month, and 3-months postoperatively. OCT (Heidelberg Engineering, Germany) was performed in all patients as part of the routine postoperative protocol at the 1-month and 3-month visits. Additionally, OCT was performed at any time within the 90-day postoperative period if a patient reported symptoms of blurred vision or if unexplained vision loss (best-corrected visual acuity ≤ 20/25) was documented.

### Definitions and grouping

In the present study, clinical IGS was defined as the presence of both: 1) Clinically evident CME confirmed by OCT, and 2) Subjective blurred vision symptoms within 90-days after uneventful PEI.^10^^,^^14^ In this study, uneventful PEI was defined as a procedure without posterior capsule rupture, vitreous loss, significant corneal edema, or other intraoperative complications. All IGS patients met the following inclusion criteria: 1) With initial diagnosis between June 2022 and January 2025, and 2) With at least one preoperative OCT scan revealing no significant macula abnormalities. The non-IGS controls were derived from patients who underwent uneventful PEI between January 2024 and January 2025 and were subsequently confirmed to be asymptomatic for blurred vision and free of CME on postoperative OCT. The exclusion criteria of both groups were as follows: participants 1) With any intraoperative iris touch or iris prolapse; 2) With any stage of DR with or without macular edema; or 3) With chronic kidney disease 3, 4, 5; or 4) With the age of ≤18-years; or 5) With any previous intraocular surgery, prior uveitis or ocular fundus diseases, including retinal vein occlusion, epiretinal membrane, preoperative prostaglandin use, vitreomacular traction, or retinal detachment.

The diagnosis of T2DM required either a physician-documented history of T2DM or current treatment with oral hypoglycemic agents (with or without insulin therapy). Hypertension was defined as a documented history of arterial hypertension or current use of at least one antihypertensive medication. Patients who underwent bilateral PEI during the enrollment period were assigned to the IGS group if IGS developed in either eye, and to the non-IGS group only in the confirmed absence of IGS in both eyes.

### Sample size and statistical analysis

All statistical analyses were performed using SPSS Statistics for Windows (version 25.0; IBM Corp). Continuous variables are presented as mean ± standard deviation, and categorical variables as counts (percentages). Normality of distribution was assessed using the Shapiro-Wilk test.

All eligible participants during the study period were included; therefore, the sample size was determined by the availability of eligible cases and controls, as is common in retrospective case-control designs. A post-hoc power analysis was conducted to evaluate whether the observed sample size was sufficient to detect the primary association between younger-onset T2DM (onset age ≤ 55.5-years) and IGS. Based on the observed proportions (IGS incidence of 15.32% in non-diabetic participants and 56.67% in younger-onset T2DM participants), with a two-sided alpha of 0.05 and using a logistic regression model, the achieved power was 89%, indicating sufficient power to detect the observed effect size (Odds Ratio [OR≈5.5]). Additionally, to address potential temporal bias arising from asymmetric recruitment windows for cases and controls, we performed a sensitivity analysis restricted to the overlapping period (January 2024 – January 2025).

Independent samples *t*-test was used for normally distributed continuous variables; Welch’s *t*-test was employed for non-normally distributed continuous variables; and Chi-square test, Fisher's exact test, or Cochran-Armitage trend test was used for categorical variables to compare. Subsequently, within the T2DM cohort, we conducted logistic regression and Receiver Operating Characteristic (ROC) curve analysis to determine the optimal predictor and its threshold for predicting IGS risk, with calculation of the Area Under the ROC Curve (AUROC). To mitigate multicollinearity, Pearson’s correlation coefficients were calculated among candidate variables. A prespecified criterion of |r| > 0.5 was used to define high collinearity. In cases where two variables exceeded this threshold, only the variable with the lower p-value from the between-group comparison was retained for subsequent multivariable modeling. We acknowledge that this data-driven selection approach may influence the perceived importance of the retained variable.

We then performed univariable logistic regression to identify potential IGS risk factors (p < 0.1) among the variables in Table 1. Statistically significant variables (p < 0.05) from this initial screening were incorporated into multivariable logistic regression models, with results expressed as adjusted ORs and corresponding 95% Confidence Intervals (95% CIs). A two-sided p-value < 0.05 was considered statistically significant for all analyses.

**Table 1:** Baseline characteristics of all recruited participants.

**Baseline characteristics**	**All** **(n** **=** **190)**	**Grouping**		**p-value**
		**IGS** **(n** **=** **39)**	**Non-IGS** **(n** **=** **151)**	
Male	84 (44.21)	13 (33.33)	71 (47.02)	0.125
Age, y	70.77±9.61	67.05±7.57	71.74±9.86	**0.006**^a^
Coexisting T2DM	79 (41.58)	20 (51.28)	59 (39.07)	0.168
Coexisting hypertension	116 (61.05)	19 (48.72)	97 (64.24)	**0.076**
Maximum systolic blood pressure, mmHg	159.82±24.75	162.05±22.54	159.39±25.25	0.669
Maximum diastolic blood pressure, mmHg	89.72±15.45	88.37±11.58	89.99±16.14	0.678
HbA1c, %	6.90±1.55	7.01±1.70	6.87±1.52	0.672
TC, mmol/L	4.49±1.13	4.51±0.99	4.48±1.16	0.926
Triglyceride, mmol/L	1.54±0.91	1.73±1.10	1.50±0.87	0.202
LDL.C, mmol/L	2.57±1.01	2.59±0.86	2.56±1.04	0.885
HDL.C, mmol/L	1.29±0.36	1.26±0.31	1.30±0.37	0.590
eGFR, mL/min/1.73m^2^	75.52±17.92	79.56±21.13	74.82±17.30	0.223
Serum creatinine, μmol/L^c^	82.99±38.73	86.61±66.72	82.21±29.60	0.717
Monocytes, × 10^9^/L	0.48±0.15	0.50±0.19	0.48±0.14	0.530
Neutrophils, × 10^9^/L	4.04±1.35	4.22±1.54	3.99±1.30	0.379
Lymphocytes, × 10^9^/L	1.96±0.70	2.00±0.75	1.95±0.69	0.706
Platelets, × 10^9^/L^c^	225.22±63.38	252.59±86.72	218.98±55.24	**0.037**^a^

	**T2DM subgroup** **(n** **=** **79)**	**Grouping**		
		**IGS (n** **=** **20)**	**Non-IGS (n** **=** **59)**	
Male	38 (48.10)	9 (45.00)	29 (49.15)	0.748
Age, y	70.24±8.15	67.20±8.04	71.27±8.00	**0.053**
T2DM duration, y^c^	13.26±8.93	18.00±10.68	11.68±7.74	**0.025**^a^
T2DM duration ≥10-years	37 (46.84)	13 (65.00)	34 (57.63)	0.562
T2DM duration ≥20-years	23 (29.11)	8 (40.00)	15 (25.42)	0.215
T2DM onset age, y	56.68±11.05	49.05±8.61	59.23±10.65	**<0.001**^b^
Coexisting hypertension	51 (64.56)	13 (65.00)	38 (64.41)	0.962
Platelets, × 10^9^/L^c^	226.38±55.97	242.79±67.39	221.00±51.22	0.142

	**Non-T2DM subgroup (n** **=** **111)**	**Grouping**		
		**IGS (n** **=** **19)**	**Non-IGS (n** **=** **92)**	
Male	46 (41.44)	4 (8.89)	42 (45.65)	**0.048**^a^
Age, y	71.15±10.54	66.89±7.26	72.03±10.92	**0.053**
Coexisting hypertension	58 (52.25)	7 (36.84)	51 (55.43)	0.140
Platelets, × 10^9^/L^c^	224.39±68.51	265.00±107.67	217.69±57.89	0.117

eGFR, Estimated Glomerular Filtration Rate; HbA1c, Glycated Hemoglobin A1c; HDL.C, High Density Lipoprotein Cholesterol; IGS, Irvine-Gass syndrome; LDL.C, Low density lipoprotein cholesterol; T2DM, Type 2 Diabetes Mellitus; TC, Total Cholesterol.

a p < 0.05.

b p < 0.001. Bold values denote p < 0.1. Data were presented as mean ± standard deviation, or n (%). Mean comparison between the two groups was performed used an independent-sample *t*-test if appropriate.

c Welch’s *t*-test was used to compare the statistical differences between the two groups. p < 0.05 was considered to be statistically significant.

## Results

### Baseline characteristics of all recruited participants

This study included 190 participants (84 males, 106 females), with one eye per subject analyzed (39 IGS eyes, 151 non-IGS eyes). The study population had a mean age of 70.77±9.61 years (range: 38–92). Among all participants, 116 (61.05%) had hypertension and 79 (41.58%) were diagnosed with T2DM. Of the 79 T2DM patients, the mean age at PEI surgery was 70.24±8.15 years, with a mean disease duration of 13.26±8.93 years and a mean onset age of 56.68±11.05 years. Comparative analysis showed that the IGS group was significantly younger (67.05±7.57 vs. 71.74±9.86 years, p = 0.006; Fig. 1A) and had higher platelet counts (252.59±86.72 vs. 218.98±55.24, p = 0.037) than the non-IGS group. No significant differences were observed in sex distribution or other clinical variables (all p > 0.05). Demographic and baseline characteristics are summarized in Table 1.

**Fig. 1:**
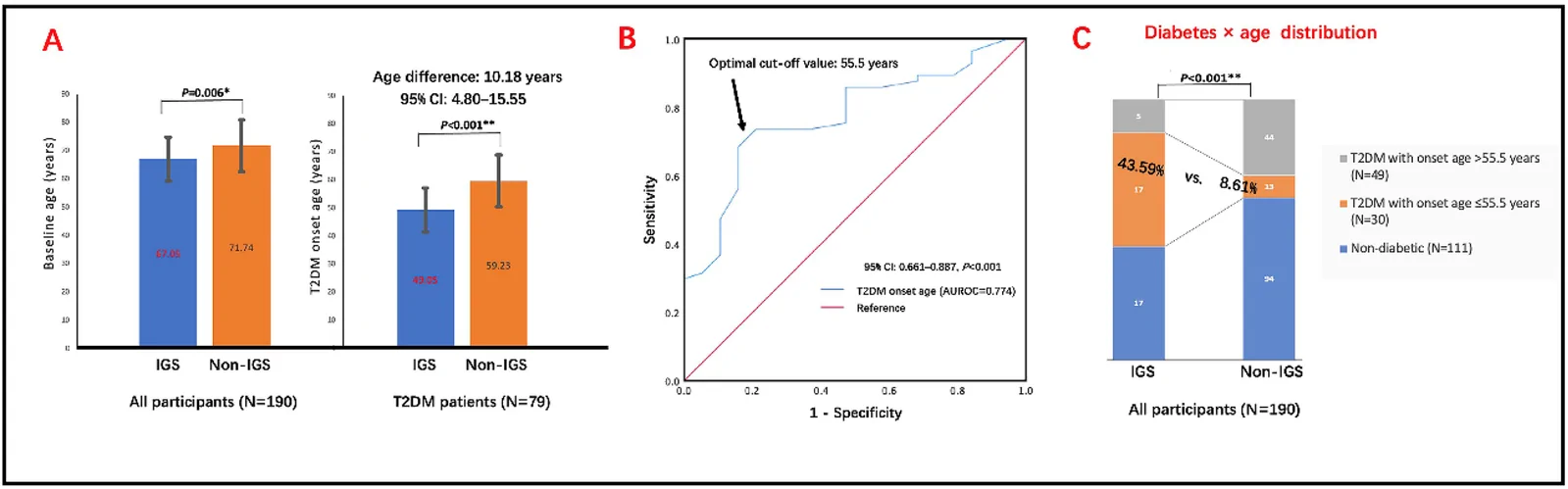
**Between-group comparisons and predictive performance of risk factors for IGS**. (A) Comparative analysis of all 190 participants revealed a significant between-group difference in age (p = 0.006, independent samples *t*‐test). Among the 79 patients with T2DM, age at diabetes onset also differed significantly between groups (p < 0.001, independent samples *t*‐test). (B) ROC analysis identified age at T2DM onset as a predictor of IGS, with an AUROC of 0.774 (95% CI 0.661–0.887, p < 0.001). At the optimal cut-off value of ≤ 55.5-years, sensitivity was 73.7% and specificity was 78.9%. (C) The Cochran-Armitage trend test indicated a significant difference in the distribution of diabetes‐age categories between IGS and non‐IGS groups (p < 0.001). AUROC, Areas Under the Receiver Operating Characteristic Curve; CI, Confidence Interval; IGS, Irvine-Gass Syndrome; OR, Odds Ratio; ROC, Receiver Operating Characteristic; T2DM, Type 2 Diabetes Mellitus. * p < 0.05. ** p < 0.001.

Although the overall prevalence of T2DM was comparable between the IGS and non-IGS groups, subgroup analysis of T2DM patients (n = 79) revealed that the IGS group had a significantly earlier age of diabetes onset (49.05±8.61 vs. 59.23±10.65 years, p < 0.001), longer diabetes duration (18.00±10.68 vs. 11.68±7.74 years, p = 0.025), and a trend toward younger baseline age (67.20±8.04 vs. 71.27±8.00 years, p = 0.053) (Table 1).

In contrast, among non-diabetic participants (n = 111), the only significant difference was in sex distribution (45% male in IGS vs. 49.15% in non-IGS, p = 0.048), with a marginal age difference (66.89±7.26 vs. 72.03±10.92 years, p = 0.053) (Table 1). Furthermore, the incidence of IGS was significantly higher in the T2DM subgroup than in the non-diabetic subgroup (27.85%vs. 15.32%, p = 0.035) (Table 2).

**Table 2:** Identification of risk factors for Irvine-Gass syndrome.

**Risk factors**	**All (n** **=** **190)**		**Grouping**				**p-value**
			**IGS (n** **=** **39)**		**Non-IGS (n** **=** **151)**		
Diabetes stratifications							**0.035**^a^
Non-diabetic	111 (100)		17 (15.32)		94 (84.68)		
T2DM	79 (100)		22 (27.85)		57 (72.15)		
Diabetes × age stratifications							**<0.001**^b^
Non-diabetic	111 (100)		17 (15.32)		94 (84.68)		
T2DM with onset age ≤ 55.5-years	30 (100)		17 (56.67)		13 (43.33)		
T2DM with onset age > 55.5-years	49 (100)		5 (10.20)		44 (89.80)		

**Risk factors**	**Univariable analysis**				**Multivariable analysis**		
	**OR**	**95% CI**	**p-value**		**OR**	**95% CI**	**p-value**
Age, per year decrease	1.050	1.013‒1.089	**0.008**^a^		1.015	0.970‒1.062	0.521
Diabetes × age stratifications							
Non-diabetic individuals	Reference				Reference		
T2DM with onset age ≤ 55.5 years	6.133	2.494‒15.086	**<0.001**^a^^,^^b^		5.499	2.104‒14.373	**0.001**^a^
T2DM with onset age > 55.5 years	0.558	0.175‒1.779	0.324		0.622	0.191‒2.030	0.432
Coexisting hypertension	0.879	0.423‒1.826	0.729		‒	‒	‒
Platelets, per 10^9^/L increase	1.008	1.002‒1.014	**0.008**^a^		1.006	1.000‒1.013	**0.052**

CI, Confidence Interval; IGS, Irvine-Gass syndrome; OR, Odds Ratio; T2DM, Type 2 Diabetes Mellitus.

a p < 0.05.

b p < 0.001. Bold values denote p < 0.1. p < 0.05 was considered to be statistically significant.

A sensitivity analysis restricting both groups to the overlapping period (January 2024–January 2025) similarly confirmed that the IGS group (n = 26) was significantly younger than the non-IGS group (n = 151; 66.12±7.97 vs. 71.74±9.86 years, p = 0.007). within the T2DM subgroup (n = 73), both baseline age (p = 0.040) and T2DM onset age (p = 0.005) were younger in IGS patients (Supplementary Table S1). In contrast, among non-T2DM subgroup (n = 104), no significant difference was observed in baseline age between the two groups (p = 0.067) (Supplementary Table S1).

### Identification of a T2DM onset age threshold for IGS in diabetic patients

Among participants with T2DM (n = 79), Pearson's bivariate correlation analysis identified significant collinearity (|r| > 0.5) between baseline age and T2DM duration (*r* = 0.741, p < 0.001). To mitigate multicollinearity in the regression model, only the variable with the lower p-value from the between-group comparison was retained for subsequent modeling; accordingly, baseline age was excluded.

In univariable logistic regression, both longer T2DM duration (OR = 1.085 per year, 95% CI 1.020–1.155, p = 0.010) and younger age at T2DM onset (OR = 1.103 per year decrease, 95% CI 1.040–1.168, p = 0.001) were significantly associated with IGS. However, in the final multivariable regression model, only younger age at T2DM onset remained an independent predictor of IGS (OR = 1.094 per year decrease, 95% CI 1.015–1.179, p = 0.019).

ROC analysis showed that age at T2DM onset predicted IGS with an AUROC of 0.774 (95% CI 0.661–0.887, p < 0.001), yielding a sensitivity of 73.7% and a specificity of 78.9% at an optimal cut-off value of ≤ 55.5-years (Fig. 1B). Furthermore, the mean age at diabetes onset in IGS patients (n = 20) was 10.18-years earlier than that in non-IGS patients (n = 59) (95% CI for the difference: 4.80–15.55 years, p < 0.001) (Fig. 1A).

A sensitivity analysis restricted to the overlapping period yielded consistent findings: T2DM onset age was significantly associated with IGS (OR = 1.086 per year decrease, 95% CI 1.021–1.156, p = 0.009), with an optimal cut-off of ≤ 57.5-years (AUROC = 0.747, 95% CI 0.607–0.887, p = 0.006; sensitivity 68.4%, specificity 76.9%).

### Predicting IGS in all recruited participants

Based on the T2DM onset age threshold through ROC analysis, all participants were stratified into three groups: non-diabetic (Group 1, n = 111), younger-onset T2DM (onset age ≤ 55.5-years, Group 2, n = 30), and older-onset T2DM (onset age > 55.5-years, Group 3, n = 49). A significant difference in IGS incidence was observed across these groups (Group 1: 15.32%; Group 2: 56.67%; Group 3: 10.20%; p < 0.001, Cochran-Armitage trend test), indicating a non-linear relationship between diabetes onset age and IGS risk (Table 2 and Fig. 1C).

Along with the diabetes-age stratification, variables from Table 1 that met the initial screening threshold (p < 0.1) ‒ namely baseline age, coexisting hypertension, and platelet count ‒ were included in the univariable regression model. The analysis identified younger age (OR = 1.050 per year decrease, 95% CI 1.013–1.089, p = 0.008), T2DM with onset age ≤ 55.5-years (OR = 6.133 vs. non-diabetic individuals, 95% CI 2.494–15.086, p < 0.001), and higher platelet count (OR = 1.008 per 10⁹/L increase, 95% CI 1.002–1.014, p = 0.008) as significant predictors of IGS. After adjusting for potential confounders, only T2DM with onset age ≤ 55.5-years remained an independent predictor (OR = 5.499 vs. non-diabetic individuals, 95% CI 2.104–14.373, p = 0.001), while platelet count showed no statistically significant association (OR = 1.006 per 10⁹/L increase, 95% CI 1.000–1.013, p = 0.052) (Table 2).

Among the 30 T2DM patients in the highest-risk group (onset age ≤ 55.5-years, Group 2), 17 were diagnosed with IGS (Fig. 1C). These high-risk IGS patients had a mean age of 66.20±8.47 years at PEI surgery, a mean T2DM duration of 20.22±10.55 years, and a mean diabetes onset age of 45.87±6.44 years.

Similarly, a sensitivity analysis restricted to the overlapping period confirmed a significant difference in IGS incidence across the three groups (11.54%vs. 43.48% vs. 8.33%, p < 0.001) and that T2DM onset ≤ 55.5-years remained an independent predictor (OR = 3.572, 95% CI 1.246–10.241, p = 0.018) (Supplementary Table S2).

## Discussion and conclusion

Our study identified younger age at T2DM onset (≤ 55.5-years) as a strong, and independent predictor of IGS following uneventful PEI. Although younger baseline patient age has been previously associated with IGS risk,^10^ subsequent regression analysis confirmed that it is specifically early-onset diabetes, rather than youth alone, that drives this risk. Furthermore, in contrast to some prior reports,^10^ we found no significant association between male sex and IGS incidence. This discrepancy may stem from our strict exclusion of patients with ocular comorbidities and surgical complications, allowing clearer isolation of systemic risk contributors. Collectively, these findings underscore that preoperative risk assessment should consider not merely the presence of diabetes, but more importantly, the age at its onset.

A key strength of our study lies in its design, which explicitly excluded participants with known ocular risk factors (such as previous retinal vein occlusion, uveitis, and CME) and surgical complications(such as intraoperative capsule rupture).^2^, ^3^, ^4^, ^5^, ^6^, ^7^^,^^15^^,^^16^ This approach enabled a focused evaluation of systemic contributors to IGS risk ‒ a methodological refinement seldom applied in prior research.^10^ While the elevated risk of IGS in diabetic patients with DR is well-documented, the risk in diabetic patients without DR has consequently been an under-characterized area of research. It was this specific knowledge gap that provided the primary rationale for our focused investigation into systemic factors, notably T2DM itself.

Recent epidemiological studies highlight a concerning threefold increase in T2DM prevalence among young adults under 40-years of age.^9^ Early-onset T2DM is clinically noteworthy due to its association with accelerated disease progression and earlier onset of complications.^9^ These insights prompted us to hypothesize that younger age at T2DM onset might represent an independent risk factor for IGS. Our ROC analysis established 55.5-years as the optimal threshold for IGS risk stratification ‒ a value notably higher than the 40-year benchmark commonly cited in general diabetes literature.^9^ This discrepancy may be explained by the delayed onset of cataract formation in individuals with early-onset T2DM, resulting in surgical intervention at an older chronological age, despite their earlier metabolic disease onset.

The observed strong association between younger-onset T2DM and IGS is likely mediated by prolonged exposure to metabolic dysregulation, chronic inflammation, and microvascular dysfunction ‒ all established mechanisms that compromise blood-retinal barrier integrity.^17^, ^18^, ^19^ In contrast to previous studies, such as those in British and New York populations, have established ocular risk factors for IGS (e.g., prior IGS in the fellow eye or pars plana vitrectomy for RRD),^20^^,^^21^ the present analysis was designed to evaluate systemic, rather than ocular, risk factors. Given the increasing global prevalence of early-onset T2DM, our findings highlight a rapidly growing patient subpopulation at significantly elevated risk of developing postoperative IGS. Incorporating the age at T2DM onset into routine preoperative assessments could thus improve risk stratification and facilitate personalized surgical management ‒ such as intensified postoperative monitoring or prophylactic interventions in high-risk individuals. This approach is further supported by previous studies indicating that, compared with placebo or steroid eye drops alone, additional prophylactic measures ‒ such as nonsteroidal anti-inflammatory drug drops, intravitreal anti-vascular endothelial growth factor agents, or subconjunctival triamcinolone acetonide ‒ may help reduce the incidence of IGS in diabetic patients.^20^^,^^22^, ^23^, ^24^, ^25^

Several limitations should be considered when interpreting the present findings. The retrospective design and single-center cohort limit the generalizability of our results. Notably, the asymmetric recruitment windows for cases (June 2022–January 2025) and controls (January 2024–January 2025) could introduce temporal bias if changes in surgical techniques, perioperative management, or diagnostic practices occurred during the extended study period. To address this concern, we performed a sensitivity analysis restricted to the overlapping period (January 2024–January 2025). Additionally, key variables like the age of T2DM onset were obtained retrospectively from medical records, which may be susceptible to inaccuracies and information bias.

The proposed mechanisms underlying the observed association, including prolonged metabolic dysregulation, chronic inflammation, and microvascular dysfunction, remain speculative. Although these pathways are well-recognized contributors to blood-retinal barrier disruption in diabetic patients, the current study does not provide direct evidence linking them specifically to IGS in the context of early-onset T2DM. As such, these mechanisms are presented as hypotheses that warrant further investigation.

Moreover, while the identified threshold of T2DM onset age (≤55.5-years) represents a clinically actionable tool for preoperative risk stratification, the optimal prophylactic or monitoring strategy for this high-risk population remains to be established. Future prospective, multiethnic studies are needed to confirm the 55.5-year risk threshold and explore the pathophysiological links between early-onset T2DM and blood-retinal barrier compromise, as well as to inform evidence-based management protocols tailored to this patient subgroup.

In conclusion, this study identifies younger age at T2DM onset (≤55.5-years) as a strong and independent risk factor for IGS following uneventful PEI. The delineation of this specific threshold provides a clinically actionable tool for preoperative risk stratification. These results underscore the critical importance of moving beyond the mere presence of diabetes and incorporating the age of its onset into the preoperative assessment. For patients with younger-onset T2DM, this threshold helps identify a high-risk subgroup that may benefit from enhanced postoperative monitoring. However, further research is needed to determine the optimal prophylactic or management strategies for this population before specific interventions can be routinely recommended.

## Abbreviations and acronyms

AUROC, Areas Under the Receiver Operating Characteristic Curve; CI, Confidence Intervals; CME, Cystoid Macular Edema; DR, Diabetic Retinopathy; IGS, Irvine-Gass Syndrome; OCT, Optical Coherence Tomography; ORs, Odds Ratios; PEI, Phacoemulsification with Intraocular lens Implantation; ROC, Receiver Operating Characteristic; T2DM, Type 2 Diabetes Mellitus.

## Declaration of generative AI and AI-assisted technologies

Not applicable.

## Statement of ethics

This study protocol received approval (n° 25,586-6–01) from the Institutional Review Board of Beijing Tsinghua Changgung Hospital and adhered to the principles outlined in the Declaration of Helsinki. Due to the retrospective design of the study, the review board granted a waiver for written informed consent. This retrospective case-control study was conducted and reported in accordance with the Strengthening the Reporting of Observational Studies in Epidemiology (STROBE) guidelines.

## Funding

No external funding was received for this study.

## Declaration of competing interest

The authors declare no conflicts of interest.
