# Supplementary material for: Earlier onset of type 2 diabetes mellitus as a risk factor for Irvine-Gass syndrome
Source: Clinics (Sao Paulo). 2026 Oct 14;81:101204. doi: 10.1016/j.clinsp.2026.101204 (PMC13693337; doi:10.1016/j.clinsp.2026.101204)
Supplement: Supplementary file 1 [file mmc1.docx]

CLINICS-D-25-02017_Supplementary Material

**Supplementary Table S1** Baseline characteristics of all recruited participants.

| **Baseline characteristics** | **All (n = 177)** | **Grouping** | | **p-value** |
| --- | --- | --- | --- | --- |
|  |  | **IGS (n = 26)** | **Non-IGS (n = 151)** |  |
| Male | 80 (45.20) | 9 (34.62) | 71 (47.02) | 0.240 |
| Age, y | 70.91±9.79 | 66.12±7.97 | 71.74±9.86 | **0.007^a^** |
| Coexisting T2DM | 73 (41.24) | 14 (53.85) | 59 (39.07) | 0.158 |
| Coexisting hypertension | 111 (62.71) | 14 (53.85) | 97 (64.24) | 0.311 |
| Maximum systolic blood pressure, mmHg | 159.52±24.81 | 160.67±21.90 | 159.39±25.25 | 0.866 |
| Maximum diastolic blood pressure, mmHg | 89.99±15.53 | 90.00±11.58 | 89.99±16.14 | 0.998 |
| Hemoglobin A1c, % | 6.84±1.49 | 6.73±1.40 | 6.87±1.52 | 0.713 |
| TC, mmol/L | 4.49±1.14 | 4.51±1.00 | 4.48±1.16 | 0.905 |
| Triglyceride, mmol/L | 1.54±0.91 | 1.78±1.15 | 1.50±0.87 | 0.164 |
| LDL.C, mmol/L | 2.57±1.02 | 2.61±0.89 | 2.56±1.04 | 0.851 |
| HDL.C, mmol/L | 1.29±0.36 | 1.24±0.31 | 1.30±0.37 | 0.494 |
| eGFR, mL/min/1.73m^2^ | 75.01±17.80 | 76.48±21.13 | 74.82±17.30 | 0.711 |
| Serum creatinine, μmol/L ^c^ | 83.92±39.17 | 93.90±66.72 | 82.21±29.60 | 0.168 |
| Monocytes, ×10^9^/L | 0.48±0.15 | 0.49±0.20 | 0.48±0.14 | 0.614 |
| Neutrophils, ×10^9^/L | 4.01±1.35 | 4.11±1.62 | 3.99±1.30 | 0.686 |
| Lymphocytes, ×10^9^/L | 1.95±0.69 | 1.96±0.71 | 1.95±0.69 | 0.903 |
| Platelets, ×10^9^/L ^c^ | 223.05±62.96 | 246.35±94.16 | 218.98±55.24 | 0.161 |
|  | **T2DM subgroup**  **(n = 73)** | **Grouping** | |  |
|  |  | **IGS (n = 14)** | **Non-IGS (n = 59)** |  |
| Male | 35 (48.10) | 6 (45.00) | 29 (49.15) | 0.672 |
| Age, y | 70.30±8.31 | 66.21±8.68 | 71.27±8.00 | **0.040^a^** |
| T2DM duration, y ^b^ | 12.50±8.54 | 16.08±11.06 | 11.68±7.74 | 0.195 |
| T2DM duration ≥ 10-years | 42 (46.84) | 8 (65.00) | 34 (57.63) | 0.974 |
| T2DM duration ≥ 20-years | 20 (29.11) | 5 (40.00) | 15 (25.42) | 0.658 |
| T2DM onset age, y | 57.49±11.07 | 49.85±9.91 | 59.23±10.65 | **0.005^a^** |
| Coexisting hypertension | 46 (64.56) | 8 (65.00) | 38 (64.41) | 0.613 |
| Platelets, ×10^9^/L | 223.89±55.70 | 235.86±72.44 | 221.00±51.22 | 0.374 |
|  | **Non-T2DM subgroup (n = 104)** | **Grouping** | |  |
|  |  | **IGS (n = 12)** | **Non-IGS (N = 92)** |  |
| Male | 45 (43.27) | 3 (25.00) | 42 (45.65) | 0.172 |
| Age, y | 71.34±10.72 | 66.00±7.45 | 72.03±10.92 | 0.067 |
| Coexisting hypertension | 57 (54.81) | 6 (50.00) | 51 (55.43) | 0.722 |
| Platelets, ×10^9^/L^b^ | 222.46±67.84 | 258.58±57.89 | 217.69±57.89 | 0.256 |

eGFR, Estimated Glomerular filtration rate; HbA1c, Glycated Hemoglobin A1c; HDL.C, High density Lipoprotein Cholesterol; IGS, Irvine-Gass Syndrome; LDL.C, Low Density Lipoprotein Cholesterol; T2DM, Type 2 Diabetes Mellitus; TC, Total Cholesterol.

^a^ p < 0.05. ** p < 0.001. Bold values denote p < 0.1.

Data were presented as mean ± standard deviation, or n (%). Mean comparison between the two groups was performed used an independent-sample *t*-test if appropriate.

^b^ Welch’s *t*-test was used to compare the statistical differences between the two groups.

p < 0.05 was considered to be statistically significant.

**Supplementary Table S2** Identification of risk factors for Irvine-Gass syndrome.

| **Risk factors** | **All (n = 177)** | | | **Grouping** | | | **p-value** |
| --- | --- | --- | --- | --- | --- | --- | --- |
|  |  |  |  | **IGS (n = 26)** | | **Non-IGS (n = 151)** |  |
| Diabetes stratifications |  | | |  | |  | **0.158** |
| Non-diabetic | 104 (100) | | | 12 (11.54) | | 92 (88.46) |  |
| T2DM | 73 (100) | | | 14 (19.18) | | 59 (80.82) |  |
| Diabetes × age stratifications |  | | |  | |  | **<0.001^b^** |
| Non-diabetic | 104 (100) | | | 12 (11.54) | | 92 (88.46) |  |
| T2DM with onset age ≤ 55.5-years | 23 (100) | | | 10 (43.48) | | 13 (56.52) |  |
| T2DM with onset age > 55.5-years | 48 (100) | | | 4 (8.33) | | 44 (91.67) |  |
| **Risk factors** | **Univariable analysis** | | | **Multivariable analysis** | | | |
|  | **OR** | **95% CI** | **p-value** |  | **OR** | **95% CI** | **p-value** |
| Age, per year decrease | 1.056 | 1.014‒1.100 | **0.009^a^** |  | 1.043 | 0.996‒1.091 | 0.072 |
| Diabetes × age stratifications |  |  |  |  |  |  |  |
| Non-diabetic individuals | Reference |  |  |  | Reference |  |  |
| T2DM with onset age ≤ 55.5-years | 4.600 | 1.655‒12.784 | **0.003**^a^ |  | 3.572 | 1.246‒10.241 | **0.018^a^** |
| T2DM with onset age > 55.5-years | 0.697 | 0.213‒2.285 | 0.551 |  | 0.804 | 0.239‒2.701 | 0.724 |
| Coexisting hypertension | 0.773 | 0.339‒1.761 | 0.540 |  | ‒ | ‒ | ‒ |

CI, Confidence Interval; IGS, Irvine-Gass syndrome; OR, Odds Ratio; T2DM, Type 2 Diabetes Mellitus.

^a^ p < 0.05. ^b^ p < 0.001. Bold values denote p < 0.1.

p < 0.05 was considered to be statistically significant.
